# Detection of Placental Proteomes at Different Uterine Positions in Large White and Meishan Gilts on Gestational Day 90

**DOI:** 10.1371/journal.pone.0167799

**Published:** 2016-12-09

**Authors:** Long Che, Mengmeng Xu, Zhenguo Yang, Shengyu Xu, Lianqiang Che, Yan Lin, Zhengfeng Fang, Bin Feng, Jian Li, Daiwen Chen, De Wu

**Affiliations:** Key Laboratory for Animal Disease Resistance Nutrition of the Ministry of Education of China, Animal Nutrition Institute, Sichuan Agricultural University, China; Universite du Quebec a Trois-Rivieres, CANADA

## Abstract

Within-litter uniformity in pigs is a major factor affecting piglet survival and growth performance. We know that Meishan (MS) gilts have higher piglet survival rate than Large White (LW) gilts because their foetal weight is less varied. To understand the molecular basis for placental nutritional transport during the late stages of gestation in LW and MS, we employed the isobaric tags for relative and absolute quantification (iTRAQ) method to investigate alterations in the placental proteomes of LW and MS gilts on gestational day 90. Investigation of foetal weight at different uterine positions revealed that the foetal and placental weights as well as the foetal concentration of glucose were significantly higher in LW gilts positioned towards the utero-tubal junction than in those positioned toward the cervix; however, no such differences were observed in MS gilts, and MS gilts had a greater uniformity in foetal weight on day 90 of gestation. Comparisons of the proteomes between placentas positioned toward the cervix and those positioned toward the utero-tubal junction identified 38 differentially expressed proteins in the two breeds. These proteins play a central role in nutrient transport and metabolism, as well as in transcriptional and translational regulation. Of particular interest is the finding that the placentas of LW gilts showed 14 differential expression of proteins mainly related to lipid transport and energy metabolism (including solute carrier family 27, mitochondrial trifunctional protein, and NADH dehydrogenase [ubiquinone] flavoprotein 2), but only 2 proteins in MS gilts. In contrast, the differentially expressed proteins in MS gilts were primarily involved in transcriptional and translational regulation (such as ribosome-sec61 and 40S ribosomal protein S23), with a few related to glucose and coenzyme transport and metabolism (including glucose transport protein and ferrochelatase). Our results revealed that placental lipid and energy metabolism might play a crucial role in the regulation of foetal weight, based on uterine position in two distinct pig breeds. These findings provide a deeper understanding of placental efficiency that can be utilized to provide a new method to enhance the efficiency of livestock production.

## Introduction

Within-litter variation in foetal weight (CVweight) is a major concern in the animal. High CV_weight_ is a common complication that has a profound impact on pre- and post-natal survival and growth rates until weaning [1–3]. Furthermore, litters with greater birth weight variation have more variable weights at weaning, which results in increased management costs due to the fact that low-birth-weight piglets need a longer time to reach the optimal slaughter weight than their larger littermates [4]. While there are probably numerous contributors to the higher proportion of low-birth-weight piglets in a litter, the factor that has attracted much attention is the efficiency of placental transport and metabolism [5,6], thus a better understanding of it can assist us in solving problem of CV_weight_.

Compared to commercial pig breeds, Chinese Meishan (MS) pigs exhibit a higher piglet survival rate, which is correlated with a lower CV_weight_ [7]. Although foetal genotype may determine placental and endometrial vascularity during the late stages of gestation, previous studies revealed that placental size, placental weight and nutrient transport efficiency are crucial factors in determining foetal weight, regardless of the foetal genotype [8]. Because an experiment in which commercial Large White (LW) breeds and MS embryos were co-transferred into the uteri of either MS or LW recipients suggested that placental efficiency sets the upper limit on foetal weight [9]. In commercial breeds, several studies investigating the relationship between intrauterine position and foetal weight have indicated that foetal weight increases linearly from the cervix to the utero-tubal junction during late gestation [10, 11]. Therefore, foetal weight differences at both sides of the uterine horn represent an important factor influencing CV_weight_ and may be associated with differences in placental nutrient transport efficiency according to uterine position [12]. Unfortunately, although it is known that MS pigs have greater uniformity in foetal weight, there is no effective research investigating the relationship between foetal weight and uterine position. In addition, information about the molecular mechanisms regulating placental nutrient transport is critical for understanding the CV_weight_ in LW and MS gilts during pregnancy, as well as how maternal nutrition and metabolic disturbances affect foetal growth.

Thus, the aim of this study was to compare the efficiency and expression of proteins involved in different physiological processes in the placenta in different uterine locations of two prolific breeds of pigs. Therefore, we focused on placental nutrient transport and metabolism at different uterine positions in pregnant LW and MS pigs by investigating differences in the expression profiles of placental proteins using isobaric tags for relative and absolute quantification (iTRAQ). Our results provide new insight into the molecular basis of placental efficiency in two distinct pig breeds with different amounts of variation in foetal weight.

## Materials and Methods

### Animal management and experimental design

The experimental protocols used in the present study were approved by the Agricultural Animal Care and Use Committee of Sichuan Agricultural University. Twelve primiparous purebred Large White (LW) gilts with an average weight of 135.54 ± 0.66 kg and the same number of Meishan (MS) gilts with an average body weight of 72.84 ± 0.66 kg were used in this study. The LW gilts were artificially inseminated twice with freshly diluted semen from the same LW boar. The MS gilts were mated with freshly diluted semen from the same MS boar. The day of the last insemination was treated as the first day of gestation. After insemination, all of the gilts were housed in individual feed stalls in the same gestation crate and fed twice daily at 07:00and 15:00 h. The feeding protocol was 2.0 kg/d from d 0 to 30, 2.4 kg/d from d 31 to 90 and 3.0 kg/d from d 91 to farrow. The diet provided to the LW and MS gilts was recommended by the National Research Council (2012) for gestational gilts.

### Measurements at slaughter

After a 12-h overnight fast on days 35, 55 and 90 of gestation, four gilts were selected randomly from each breed for weighing and anaesthetization via intramuscular injection of Zoletil 50 (Zoletil 50 Vet, Virbac, France) at a dose of 0.1 mg/kg body weight. After the animals were deeply anaesthetised, the abdomen was opened and the uterus was immediately collected. The uterine horns were separated from the broad ligament to collect all of the foetuses. The length and weight of each foetus were recorded. The weights of the foetuses and placentas located in each uterine horn from the cervix to the utero-tubal junction were recorded on days 35, 55 and 90 of gestation. On day 90 of gestation, blood samples were collected from the foetuses located at the cervix and utero-tubal junction. After centrifugation of the blood samples at 3500 × g for 10 min, the isolated serum samples were collected and stored at -20°C. Gestational day 90 was chosen to collect the placentas positioned toward the cervix or the utero-tubal junction because it is a crucial time point of foetal weight variation at which placental nutrient transport and metabolism become limiting factors for foetal growth.

### Serum biochemical measures

Foetal serum IGF-1 concentrations were determined using enzyme-linked immunosorbent assay kits (R&D Systems Inc., Minneapolis, MN, USA) with a sensitivity of 0.01 ng ⁄ml according to the manufacturer’s instructions. Glucose concentrations were measured using a standard glucose oxidase assay kit (Nanjing Jiancheng Bioengineering Institute, Nanjing, China) with a sensitivity of 0.01 ng ⁄ml.

### Protein extraction

Placental samples were obtained from 4 pregnant LW and MS gilts on gestational day 90 before protein extraction. The placental sampling points were located towards the cervix and the utero-tubal junction in both breeds. Each point consisted of two biological replicates and each biological replicate sample was a pooled sample from four randomly selected placental samples, supplied by two gilts at the same uterine site (each gilt provided two placenta samples, one from the right and left of the uterus). Protein extraction was performed as previously described [13]. Briefly, each pooled placental sample was ground into powder using liquid nitrogen and lysed in lysis buffer consisting of 7 M urea, 4% CHAPS, 40 mM Tris-HCl, 1 mM PMSF and 2 mM EDTA. After sonication, the suspension was centrifuged at 4°C and 30 000 g for 15 min. The supernatant was precipitated with 10% (v/v) trichloroacetic acid (TCA) and 90% ice-cold acetone at −20°C overnight. The precipitate was collected through centrifugation and resuspended in lysis buffer. Then, the suspension was re-sonicated and centrifuged at 4°C and 30 000 g for 15 min. Subsequently, 55 mM IAM was added to the supernatant to block the cysteines before incubation for 1 h in the dark. After reduction and alkylation, the proteins were precipitated by adding a 55 × volume of chilled acetone for 2 h at -20°C. After centrifugation, the pellet was air-dried for 5 min, dissolved in 500 μL of 0.5 M TEAB (Applied Biosystems, Milan, Italy), and sonicated at 200 W for 15 min. Finally, the samples were centrifuged at 4°C and 30 000 g for 15 min, and the supernatant from each group was prepared for subsequent analysis.

### iTRAQ labelling

Trypsin Gold (Promega, Madison, WI, USA) was added to each sample (total protein: 100μg) at a 1:30 ratio, and the samples were digested for 16 hours at 37°C. After trypsin digestion, the peptides were dried, reconstituted in 0.5 M TEAB and processed according to the manufacturer’s protocol for 8-plex iTRAQ (Applied Biosystems). All of the protein samples were labelled with iTRAQ tags as follows: tag 115 for the placentas located at the utero-tubal position in LW gilts; tag 116 for the placentas located at the cervical position in LW gilts; tag 117 for the placentas located at the utero-tubal position in MS gilts; and tag 118 for the placentas located at the cervical position in MS gilts.

### SCX fractionation

First, the iTRAQ-labelled peptide mixtures werereconstitutedin4 mL of buffer A (25 mM NaH_2_PO_4_ in 25% ACN, pH 2.7) using a LC-20AB HPLC Pump system (Shimadzu, Kyoto, Japan) and loaded onto a 4.6×250 mm Ultremex SCX column containing 5-μm particles (Phenomenex, Torrance, CA, USA). Second, the peptides were eluted at a flow rate of 1 mL/min with a gradient of buffer A for 10 min, 5–60% buffer B (25mM NaH_2_PO_4_, 1 M KCl in 25% ACN, pH 2.7) for 27 min, and 60–100% buffer B for 1 min. A total of 20 fractions were collected from the eluted peptides, and each fraction was desalted with a Strata X C18 column (Phenomenex, Torrance, CA, USA) and vacuum-dried.

### Mass spectrometry

Each fraction was resuspended in buffer A (5% ACN, 0.1% FA) and centrifuged at 20000 g for 10 min. The average final peptide concentration was approximately 0.5 μg/μl. Then, we loaded 10μl of supernatant onto a 2cm C18 trap column in an LC-20AD Nano HPLC pump system using an auto sampler. The samples were loaded at 8 μl/min for 4 min and eluted at 300 nL/min for35 min with a 2% to 35% gradient of solution B (95% acetonitrile, 0.1% formic acid). The samples were separated with a 5-min linear gradient to 60% solution B, maintained at 80% solution B for 2 min, and finally returned to 5% solution B over 1 min. Data acquisition was performed with a Triple TOF 5600 System (AB SCIEX, Concord, ON) fitted with a Nanospray III source (AB SCIEX, Concord, ON) and a pulled quartz tip as the emitter (New Objectives, Woburn, MA). Data were acquired at 250 ms intervals, and as many as 30 product ion scans were collected if a threshold of 120 counts per second (counts/s) was exceeded with a 2^+^ to 5^+^ charge-state and a 15-s dynamic exclusion setting.

### Validation of proteins of differential abundance

Real-time PCR (RT-PCR) was used to confirm differences in the levels of mRNAs corresponding to the eight placental proteins exhibiting differential abundance. Total RNA was isolated from tissues using TRIzol (Invitrogen, Carlsbad, CA, USA) according to the manufacturer's instructions. Agarose gel electrophoresis was used to evaluate the RNA quality. Reverse transcription was performed with a high-capacity cDNA reverse transcription kit (TaKaRa Biotechnology, Dalian, China). All mRNA expression levels were analysed via RT-PCR using a PrimeScript™ RT reagent kit (TaKaRa, Biotechnology, Dalian, China) and an Applied Biosystems ABI 7900HT Fast Real-Time PCR System (Foster City, CA, USA). The PCR conditions were as follows: 95°C for 60 s followed by 40 cycles of 95°C for 5 s and 72°C for 30 s. All of the real-time PCR experiments were performed in triplicate. Agarose gel electrophoresis was used to confirm product size. β-actin was used as the housekeeping gene. The primer sequences used are listed in S1 Table. In addition, high-confidence peptides of the target proteins exhibiting rich product ion spectrum were selected for multiple reaction monitoring (MRM). The proteins with a threshold of >1.2- or <0.83—fold were considered differentially expressed proteins (Liu et al., 2015; Zhang et al., 2015) because of iTRAQ is comparatively of high validity. Therefore, we chose the 20 differentially expressed proteins (The proteins with a threshold of >1.2- or < 0.83, S2 Table) from iTRAQ to validate with MRM. The MRM analysis are described in S3 Table.

### Statistical analysis

For the reproductive performance results, serum biochemical index data were analysed using Student’s t-test in SPSS software (v. 19.0 for Windows; IBM SPSS Company, Chicago, IL, USA). All of the experimental data are presented as the means ± SEM. CV_weight_ data were analysed using theχ^2^-test in SPSS. AP value <0.05 was considered statistically significant. For protein quantitation, the quantitative protein levels were weighted and normalized to the median levels in Mascot; the proteins containing at least two unique spectra were considered for analysis. Proteins with a threshold of >1.3- or <0.77-fold difference in abundance with P values <0.05 were considered differentially expressed proteins. The relative differences in the levels of target genes in placental tissues were determined using the 2^-ΔΔCt^ method [14]. Results with P < 0.05 were considered significant.

## Results

### Reproductive performance of LW and MS gilts

**Fig 1:**
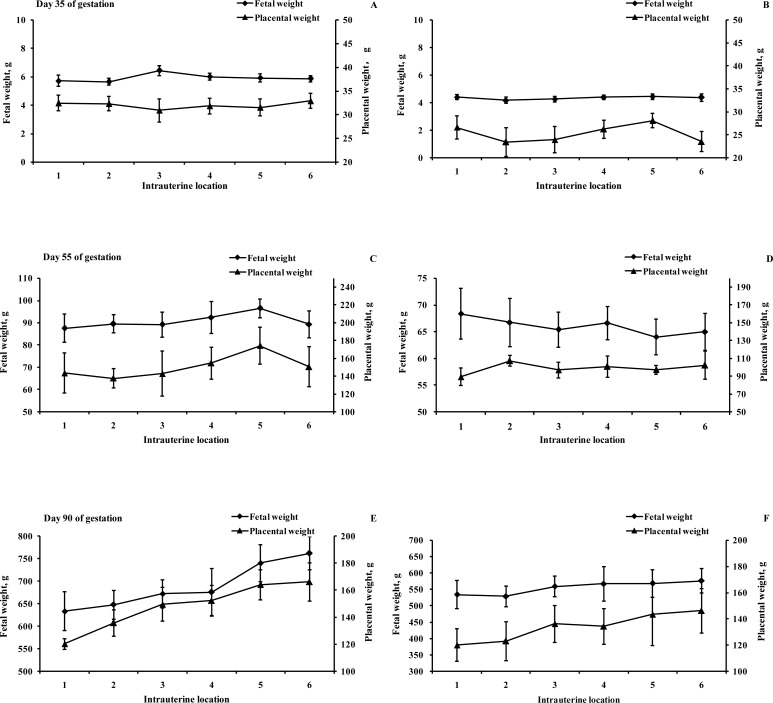
Distribution of fetal and placental weights (g) within the uterine horn on day 35, 55 and 90 of gestation. Fetal and placental weights of Large White gilts in relation to fetal rank within the uterine horn (A, C, E); Fetal and placental weights of Meishan gilts in relation to fetal rank within the uterine horn (B, D, F). Fetal positions within the uterine horn were numbered from cervix (1) to tubal (6).

Foetal weight and length as well as variations in foetal weight and length are shown in Table 1. Foetal weight was not significantly different between the two breeds on gestational day 35 (P>0.05). However, compared with MS gilts, the foetal weights of LW gilts were higher on gestational days 55 and 90 (P<0.05). There was no difference in foetal length between the breeds on gestational days 35 and 55 (P>0.05); however, the LW breed had a higher foetal length on gestational day 90 (P<0.05). On gestational days 35, 55 and 90, the CV_weight_ was significantly higher in LW gilts than in MS gilts (P<0.05). As shown in Fig 1, it is noteworthy that the foetal and placental weights of LW gilts increased from the cervix to the utero-tubal junction, and the foetal and placental weights of LW gilts were significantly higher for the foetuses positioned toward the utero-tubal junction than for the foetuses positioned toward the cervix on gestational day 90 (P<0.05). These results indicate that the effects of location within the uterine horn on foetal piglet weight are more notable during late gestation. However, foetal weight did not differ consistently according to position within the uterine horn among MS gilts on gestational day 90.

**Table 1:** Fetal weight, fetal length, variation in fetal weight and length within a litter in Large White and Meishan gilts.

Items	Days of gestation	LW	MS	P-value
Fetus weight, g	35	5.92 ± 0.39	4.33 ± 0.23	0.014
	55	86.07 ± 3.56	61.58 ± 1.41	<0.01
	90	647.18 ± 31.12	507.71 ± 24.01	<0.01
Fetal length, cm	35	3.98 ±0.10	3.58 ± 0.12	0.038
	55	11.85 ± 0.28	10.98 ± 0.28	0.729
	90	23.46 ± 0.33	21.82 ± 0.28	<0.01
CV_weight_, %	35	8.1 ± 0.8	4.9 ± 0.7	0.027
	55	14.9 ± 1.0	9.4 ± 1.6	0.048
	90	14.6 ± 0.9	10.6 ± 0.6	0.011
CV_length_, %	35	6.3 ± 1.4	4.9 ± 0.7	0.395
	55	5.0 ± 0.8	3.8 ± 0.7	0.290
	90	6.4 ± 0.6	5.2 ± 0.6	0.185

CV_weight_: within-litter variation in fetal weight; CV_length_: within-litter variation in fetal length; LW: Large White gilts; MS: Meishan gilts.

### Serum glucose and IGF-1concentrations

**Fig 2:**
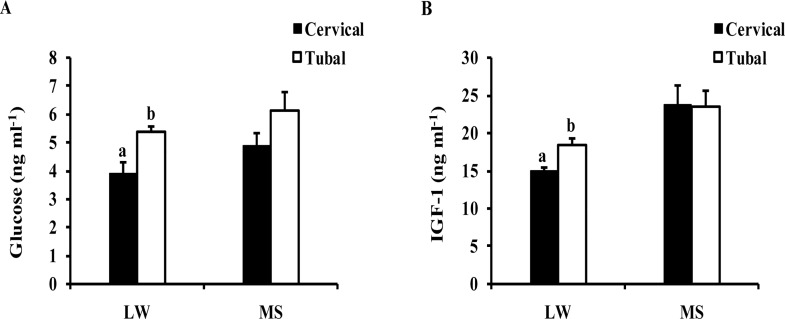
Serum parameters of the fetuses from different uterine position on day 90 of gestation. Cervical: the fetuses at the position toward the cervix; Tubal: the fetuses at the position toward the utero-tubal junction; LW: Large White gilts; MS: Meishan gilts.

For LW gilts, the glucose and IGF-1 concentrations of the foetuses located towards the cervix were significantly lower than those of the foetuses located towards the utero-tubal junction (P<0.05). However, there was no significant difference in the glucose or IGF-1 concentrations between these two locations in MS gilts (P>0.05) (Fig 2).

### Placental proteomes

In this study, a total of 2047 proteins were identified in the placentas positioned towards the cervix and the utero-tubal junction, with a false discovery rate of 1% (S4 Table). A total of 38 proteins were differentially expressed between the placentas located at the cervix and those located at the utero-tubal junction in both MS and LW gilts (Table 2). According to their biological function, these differentially expressed proteins were classified into seven groups: (1) carbohydrate transport and metabolism; (2) amino acid transport and metabolism; (3) lipid transport and metabolism; (4) energy production and conversion; (5) inorganic ion transport and metabolism; (6) coenzyme transport and metabolism; and (7) transcriptional and translational regulation.

**Table 2:** Differentially expressed proteins in the placenta in Large White and Meishan gilts.

Accession number	Protein name	Protein score	LW		MS	
			Cervix	Tubal	Cervix	Tubal
Carbohydrate transport and metabolism						
gi|1956	Glucose transport protein	569	1.00^a^	1.36^b^	-1.12^b^	-1.50^a^
gi|311259466	Putative hydroxypyruvate isomerase isoform X1	159	1.00^b^	-1.41^a^	-1.22	1.05
gi|194033484	Plasma alpha-L-fucosidase	167	1.00^b^	-1.52^a^	-1.69^a^	-1.09^b^
gi|350579297	Phosphoglucomutase-like protein 5	634	1.00^b^	-1.31^a^	-1.21	0.90
gi|937575546	Fucosidase alpha-L- 1	163	1.00	-1.21	-1.02^b^	-1.36^a^
gi|753703824	Galactosidase, beta 1	5025	1.00	-1.01	-1.60^a^	-1.30^b^
Amino acid transport and metabolism						
gi|456753185	Proline dehydrogenase (oxidase) 1	69	1.00^a^	1.55^b^	1.11	1.06
gi|345091045	Cationic amino acid transporter 4	366	1.00^a^	1.33^b^	-1.06	-1.15
gi|297591979	Ornithine amino transferase	258	1.00	1.18	-1.02^b^	-1.40^a^
gi|927096340	Creatine kinase U-type, mitochondrial isoform X1	867	1.00^a^	1.39^b^	1.09	1.00
Lipid transport and metabolism						
gi|927129928	Retinol dehydrogenase 16	2649	1.00^a^	1.44^b^	1.62	1.49
gi|927170871	3-hydroxybutyrate dehydrogenase type 2	115	1.00	-1.04	-1.54^a^	-1.14^b^
gi|47522658	Corticosteroid 11-beta-dehydrogenase isozyme 2	1202	1.00^a^	1.48^b^	-1.08	-1.15
gi|27066006	20beta-Hydroxysteroid Dehydrogenas	1247	1.00^a^	1.46^b^	-1.05	-1.19
gi|523580064	Long-chain acyl-CoA synthetase ACSL	90	1.00^a^	1.38^b^	-1.12	-1.40
gi|417515459	Solute carrier family 27	416	1.00^a^	1.30^b^	-1.14	-1.10
gi|2494384	Liver carboxylesterase	879	1.00^b^	-1.79^a^	2.13	2.13
gi|7387634	Mitochondrial trifunctional protein	3812	1.00^a^	1.59^b^	1.58	1.37
gi|545854135	Isopentenyl-diphosphate Delta-isomerase 1 isoform X1	291	1.00^a^	1.31^b^	1.39	1.15
Energy production and conversion						
gi|353819	Cytochrome b5	1250	1.00^a^	1.30^b^	-1.12	-1.08
gi|545882274	V-type proton ATPase 116 kDa subunit a isoform 4	358	1.00^a^	1.31^b^	1.22	1.25
gi|343403802	Delta(24)-sterol reductase	461	1.00^a^	1.47^b^	-1.02	-1.00
gi|223022	Adrenodoxin	117	1.00^a^	1.57^b^	1.28	-1.01
gi|545835136	NADH dehydrogenase [ubiquinone] flavoprotein 2	1107	1.00^a^	1.35^b^	1.18	1.22
gi|122138098	Aldehyde dehydrogenase	930	1.00^b^	-1.43^a^	-1.14	-1.13
gi|2497785	NADP-dependent malic enzyme	562	1.00	1.17	1.12^b^	-1.18^a^
Inorganic ion transport and metabolism						
gi|346421372	Ferritin light chain	237	1.00^b^	-1.41^a^	-2.44^a^	-1.84^b^
gi|927207348	Bifunctional 3'-phosphoadenosine 5'-phosphosulfate synthase 2	471	1.00	1.08	1.35^a^	2.23^b^
Coenzyme transport and metabolism						
gi|281427372	Ferrochelatase	796	1.00	1.08	-1.20^b^	-1.57^a^
gi|148234672	Porphobilinogen deaminase	88	1.00^a^	1.30^b^	1.07^b^	-1.35^a^
gi|335284411	Nicotinate-nucleotide pyrophosphorylase	112	1.00^b^	-1.35^a^	-1.15	-1.41
Transcriptional and translational regulation						
gi|675970388	Translating Mammalian Ribosome-sec61 Complex	896	1.00^a^	1.41^b^	1.14	-1.02
gi|6174950	60S ribosomal protein L15	118	1.00^a^	1.30^b^	-1.53	-1.30
gi|347658980	60S ribosomal protein L36	271	1.00^b^	-1.37^a^	-1.58	-2.03
gi|675970363	Translating Mammalian Ribosome-sec61 Complex	97	1.00	1.28	-1.04^b^	-1.36^a^
gi|346986341	Ribosome maturation protein SBDS	148	1.00	1.13	-1.02^b^	-1.34^a^
gi|62287175	40S ribosomal protein S23	251	1.00^a^	1.34^b^	1.32^b^	-1.10^a^
gi|89573899	Ribosomal protein L18	250	1.00	1.00	-1.65^a^	1.01^b^

Proteins with a threshold of >1.3- or <0.77-fold difference in abundance with P values <0.05. LW: Large White gilts; MS: Meishan gilts; Cervical: the fetuses at the position toward the cervix; Tubal: the fetuses at the position toward the utero-tubal junction.

### Carbohydrate transport and metabolism

A total of six differentially expressed proteins were related to carbohydrate transport and metabolism. Proteins related to carbohydrate transport included glucose transport protein, and proteins related to carbohydrate metabolism included putative hydroxypyruvate isomerase, alpha-L-fucosidase, phosphoglucomutase-like protein 5, fucosidase alpha-L-1 and galactosidase, beta 1. For LW gilts, the expression of glucose transport protein was higher in the placentas positioned towards the utero-tubal junction than in those positioned at the cervix (P<0.05); however, the proteins associated with carbohydrate metabolism were more abundant in the placentas positioned towards the cervix than in those positioned towards the utero-tubal junction (P<0.05). In contrast, in MS gilts, the expression of glucose transport protein and fucosidase alpha-L-1 was higher in the placentas positioned at the cervix than in those positioned at the utero-tubal junction (P<0.05), while the expression of fucosidase alpha-L-1 and galactosidase, beta 1 was the opposite (P<0.05).

### Amino acid transport and metabolism

The differentially expressed proteins involved in amino acid transport and metabolism were proline dehydrogenase (oxidase) 1, cationic amino acid transporter 4, ornithine amino transferase and creatine kinase U-type. In LW gilts, the expression of proline dehydrogenase (oxidase) 1, cationic amino acid transporter 4 and creatine kinase U-type were higher in the placentas positioned towards the utero-tubal junction than in those positioned towards the cervix (P<0.05). In MS gilts, the abundance of ornithine amino transferase was lower in the placentas positioned towards the utero-tubal junction than in those positioned towards the cervix (P<0.05).

### Lipid transport and metabolism

It is important to remember that lipid transport and metabolism in a placenta play an important role in placental efficiency. In LW gilts, interestingly, a total of nine differentially expressed proteins were related to lipid transport and metabolism. Compared with the proteins in the placentas positioned towards the cervix, the abundance of all the proteins was higher in the placentas positioned at the utero-tubal junction, with the exception of 3-hydroxybutyrate dehydrogenase and liver carboxylesterase. Among these proteins, solute carrier family 27 and lipid metabolic enzymes are of great importance for lipid transport and metabolism. However, this trend was different in LW gilts, as our results only identified 3-hydroxybutyrate dehydrogenase type 2 as being significantly more abundant in the placentas positioned towards the utero-tubal junction than in those positioned towards the cervix in MS gilts (P<0.05).

### Energy production and conversion

The expression of differentially expressed proteins related to energy metabolism was affected by uterine position and breed. Differentially expressed proteins such ascytochrome b5, V-type proton ATPase 116 kDa subunit, delta(24)-sterol reductase, adrenodoxin and NADH dehydrogenase [ubiquinone] flavoprotein 2 were higher in the placentas located at the utero-tubal junction than in those located at the cervix in LW gilts. However, while the expression of NADP-dependent malic enzyme was affected by uterine position in MS gilts, the differences in the abundance of the other proteins related to energy metabolism were not significant.

### Inorganic ion transport and metabolism

In LW gilts, the placentas positioned at the utero-tubal junction exhibited a higher abundance of ferritin light chain (P<0.05). In MS gilts, the abundance of ferritin light chain and bifunctional 3'-phosphoadenosine 5'-phosphosulfate synthase 2 were increased in the placentas located at the uterine tubal junction compared with those located at the cervix (P<0.05).

### Coenzyme transport and metabolism

The expression of porphobilinogen deaminase, which is related to coenzyme transport and metabolism, was affected by the position of the placenta in the uterus. In particular, its expression was higher in the placentas positioned at the utero-tubal junction. The expression of nicotinate-nucleotide pyrophosphorylase was lower in the placentas located at the cervix than in those positioned toward the utero-tubal junction in LW gilts (P<0.05). However, the abundance of ferrochelatase and porphobilinogen deaminase was higher in the placentas positioned at the cervix than in the placentas positioned at the utero-tubal junction in MS gilts (P<0.05).

### Transcriptional and translational regulation

In LW gilts, there were four differentially expressed proteins related to transcriptional and translational regulation. Compared with the placentas located at the cervix, the expression of translating mammalian ribosome-sec61, 60S ribosomal protein L15 and 40S ribosomal protein S23 were increased in the placentas located at the uterine tubal junction (P<0.05); however, 60S ribosomal protein L36showed the opposite expression pattern. In contrast, MS gilts exhibited higher expression of translating mammalian ribosome-sec61, ribosome maturation protein SBDS and 40S ribosomal protein S23 in the placentas positioned at the cervix than in those positioned at the utero-tubal junction; however, the abundance of ribosomal protein L18 was higher in the placentas located at the utero-tubal junction than in the placentas located at the cervix (P<0.05).

### Validation of proteins of differential abundance

**Fig 3:**
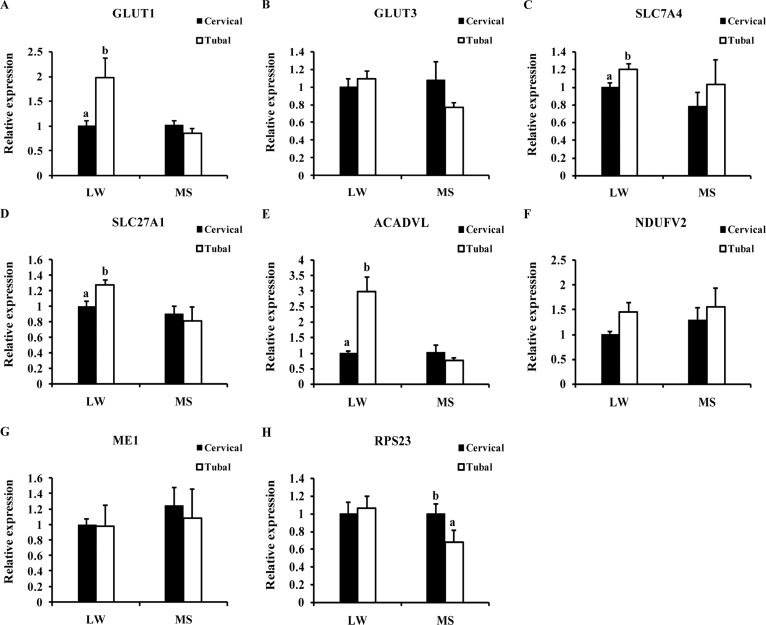
RT-PCR validation of eight proteins of differential abundance from the placenta from different uterine position at the mRNA level. Cervical: the fetuses at the position toward the cervix; Tubal: the fetuses at the position toward the utero-tubal junction; LW: Large White gilts; MS: Meishan gilts. GLUT1: Glucose transporter 1; GLUT3: Glucose transporter 3; SLC7A4: Cationic amino acid transporter 4; SLC27A1: Solute carrier family 27; ACADVL: Long-chain acyl-CoA synthetase ACSL; NDUFV2: NADH dehydrogenase [ubiquinone] flavoprotein 2; ME1: NADP-dependent malic enzyme; RPS23: 40S ribosomal protein S23.

Eight differentially expressed proteins involved in carbohydrate transport and metabolism (GLUT1 and GLUT3), amino acid transport and metabolism (SLC7A4), lipid transport and metabolism (SLC27A1 and ACADVL), energy production and conversion (NDUFV2 and ME1) or transcriptional and translational regulation (RPS23) were selected. The levels of these proteins related to nutrient transport and metabolism were consistent with their mRNA expression levels (Fig 3). In addition, although MRM analysis successfully detected 10 differentially expressed proteins from iTRAQ (S5 Table), hardly any of these proteins were detected in RT-PCR except Long-chain acyl-CoA synthetase ACSL (ACADVL). ACADVL is mainly involved in lipid transport and metabolism and its expression was consistent in iTRAQ and MRM. Several others proteins were primarily related to carbohydrate transport and metabolism, energy production and conversion, inorganic ion transport and metabolism and transcriptional and translational regulation. For example, fucosidase alpha-L- 1 is involved in carbohydrate transport and metabolism, and was significantly up-regulated in the utero-tubal end of the placenta in iTRAQ (ratio >1.3, P<0.05) and MRM (P<0.05). In LW gilts, the up-regulation of mitochondrial tri-functional proteins involved in lipid transport and metabolism in the utero-tubal end of the placenta were consistent in iTRAQ and MRM. Although there were no significant differences in the expression of some proteins in the different uterine locations using MRM, the log ratios of the quantitative data of the 10 target proteins from MRM were significantly positively correlated with those from iTRAQ (S1 Fig). These results further corroborated our belief that differences occur in the placental efficiency of two distinct pig breeds.

## Discussion

In pigs, it was interesting to note that foetal growth is significant after d 80 of gestation [11]. Thus, variation in foetal weight primarily arises late in gestation. In this study, we found greater uniformity in the weights of foetuses and piglets from MS gilts than LW gilts on gestational day 90. This is not surprising, as this phenomenon has previously been reported in MS pigs [15,16]. Interestingly, the foetal weight of LW gilts increased linearly from the cervix to the utero-tubal junction; however, there was no consistent relationship between foetal weight and position within the uterine horn in the MS breed. In fact, a previous work has demonstrated a similar relationship between foetal weight and uterine position in commercial breeds [10]. Unfortunately, while placental efficiency during late gestation has been regarded as a major factor influencing foetal growth, no studies have focused on MS gilts or on the molecular mechanisms underlying the different foetal weight variations between MS and LW gilts. Therefore, to our knowledge, this is believed to be the first study to describe the differences in the placental proteomes of foetuses according to uterine location and weight.

Convincing data have revealed that litter homogeneity is affected by foetal genotype, which can contribute to different placental and endometrial vascularity development strategies during late gestation [17]. There is evidence that the MS placenta is smaller at farrowing while the vascular density progressively increases in late gestation. In contrast, although the placental size is higher in LW gilts, the vascular density remains constant [9]. Therefore, the limited placental weight or surface area in MS gilts decreases conceptus competition for uterine space, resulting in greater uniformity in foetal weight [4]. Unfortunately, the mechanism responsible for this phenomenon is unknown. To obtain insight into the mechanism linking variations in foetal weight to different uterine locations, the placentas of LW and MS gilts were analysed for differentially expressed proteins.

Current proteomic approaches facilitate the analysis of protein abundance, thus providing an efficient tool for placental nutrient transport and metabolism research in pigs [18]. In the present study, an iTRAQ-based quantitative proteomics approach was used to investigate the variability in the placental proteome based on uterine position. Using proteomic technology, we identified a total of 38 proteins in LW and MS gilts that were differentially expressed depending on the position of the placenta within the uterus: 28 proteins were identified in LW gilts and 15 proteins were identified in MS gilts. These proteins were mainly found to be involved in the transport and metabolism of nutrients, including carbohydrates, amino acids, lipids, inorganic ions and coenzymes, as well as in energy production. In LW gilts, the differentially expressed proteins were primarily involved in nutrient transport and metabolism, especially lipid and energy metabolism. It is worth noting that the expression of most of the proteins related to nutrient metabolism was higher in the placentas located towards the uterine tubal junction than in those located towards the cervix. This trend was consistent with the differences in foetal weight. However, in MS gilts, the differentially expressed proteins mainly participated in transcriptional and translational regulation, with a few proteins being related to nutrient metabolism.

Placental transport and metabolism are major factors involved in foetal nutrition and metabolism [19]. Previous studies have indicated that the foetal overgrowth observed in women with type 1 diabetes is associated with increased glucose transporter activity and glucose transport protein (GLUT) expression [20]. GLUT1 and GLUT3 are the primary glucose transporters expressed in the placenta [21]. In our study, glucose transport protein and GLUT1 mRNA were expressed at higher levels in the placentas positioned towards the uterine tubal junction than in those positioned towards the cervix in LW gilts, thus changing the concentration of glucose received by the foetus. Glucose is the primary energetic material used for foetal growth [22], as feeding glucose to sows during late gestation increased the birth weights of live-born piglets [23]. Previous studies have indicated that all nutrient demands are supplied via placental transfer from the mother to the foetus because foetal organs do not participate in any nutrient metabolism [24]. Therefore, changes in placental glucose supply directly contribute to altered foetal growth [25]. In contrast, although glucose transport protein was expressed at higher levels in the placentas positioned towards the cervix in MS gilts, this difference did not affect the foetal concentration of glucose. We speculate that the glucose concentration was similar in the placental vasculature at different uterine positions.

Glucose is produced through the metabolism of carbohydrates, amino acids and fatty acids [26,27]. The differences in glucose concentration depending on uterine position reflect the complex state of placental nutrient transport and metabolism. Several studies have shown that the maternal lipids stored during early gestation function as an oxidative substrate late in gestation, contributing to a decrease in the maternal demand for glucose [28]. Consistent with the above theories, our results showed a dramatic change in lipid transport and metabolism in LW gilts. Nine of the differentially expressed proteins identified were classified into the lipid transport and metabolism category; interestingly, seven of these proteins were up-regulated in the placentas located towards the uterine tubal junction. Long-chain acyl-CoA synthetase (ACSL) is necessary for long acyl-CoA ester production, phospholipid remodelling, and fatty acid synthesis, including the synthesis of long chain fatty acids (LCFAs) [29]. The transport of LCFAs from the maternal plasma is crucial for foetal growth and development because LCFA synthesis in the foetus is minimal [30]. The expression of ACADVL from iTRAQ was further confirmed by MRM, suggesting it may play a role in the lipid transport and metabolism of placenta. Fatty acid transport proteins (such as SLC27A1) are required in biological membranes to promote the cellular uptake of LCFA and to meet the increased nutrient demand of the foetus [31]. In addition, differentially expressed proteins are involved in the regulation of placental growth and foetal heath. For example, mitochondrial trifunctional protein (HADHA) catalyses long-chain fatty acid oxidation and provides plenty of energy for the placenta and the foetus [32]. Retinol dehydrogenase participates in the conversion of retinol to retinoic acid, which is an important developmental signalling molecule that contributes to the proliferation and differentiation of the epithelium [33]. Corticosteroid 11 beta-dehydrogenase (11-DH) regulates cortisol metabolism within the placenta and catalyses the conversion of cortisol to the inactive metabolite cortisone. It is apparent that inappropriate exposure to high levels of cortisol may interfere with the normal trajectory of foetal development [34]. As a result, the foetal weights of LW gilts were greater at the uterine tubal junction than at the cervix, a finding that was attributed to higher placental efficiency and more sufficient nutrient supply at the uterine tubal junction. The expression of proteins related to amino acid transport and metabolism, such as cationic amino acid transporter 4 and proline dehydrogenase (oxidase) 1, followed the same trend. The placentas located at the utero-tubal junction experienced enhanced transport and metabolism of amino acids, which likely promoted energy production and nutrient transport from the mother to the foetus. However, we did not find any significant differences in the expression of these proteins between different uterine positions in MS gilts. This result supports the observed differences in foetal weight; unfortunately, the exact mechanism underlying these differences in foetal weight remains to be elucidated.

Energy is required for a variety of physiological processes in the foetus, including nutrient transport, cell motility, and biosynthetic pathways; thus, placental energy metabolism plays a crucial role in foetal development [35]. In the present study, most of the differentially expressed proteins identified in the energy metabolism categories were up-regulated in the placentas located at the uterine tubal junction in LW gilts. Of these proteins, NADH dehydrogenase [ubiquinone] flavoprotein 2 (NDUFV 2) is an important subunit involved in the production of ATP and oxidative stress within the mitochondria, regarded as the “powerhouse” of the cell. Bénit et al. (2003) confirmed that a decrease in NDUFV2 protein content led to a decrease in complex I activity [36]. Higher levels of these proteins in the placentas located at the utero-tubal junction could enhance anti-oxidative reactions and promote the generation of ATP. Furthermore, the increased abundance of proteins related to energy metabolism, including cytochrome b5, V-type proton ATPase 116 kDa subunit a isoform 4, delta(24)-sterol reductase and adrenodoxin, was observed in the placentas positioned towards the utero-tubal junction, indicating an increased capacity for ATP synthesis to meet the needs of nutrient transport [37,38]. In contrast, in MS gilts, we found that the expression of only NADP-dependent malic enzyme was up-regulated in placentas located at the cervix. Although NADP-dependent malic enzyme is believed to play an important role in the metabolism of glutamine for energy production in rapidly proliferating tissues [39], changes in the levels of this particular protein cannot affect foetal weight due to insufficient substrate levels.

Although our results revealed several differentially expressed proteins in placentas according to uterine position in MS gilts, these proteins were mainly related to transcriptional and translational regulation, with a few being involved in nutrient transport and metabolism. The expression of proteins involved in transcriptional and translational regulation (such as translating mammalian ribosome-sec61 complex, ribosome maturation protein SBDS and 40S ribosomal protein S23) was down-regulated in the placentas located at the uterine tubal junction, indicating a decreased capacity for protein synthesis to maintain placental function and integrity [40–42]. Convincing data have revealed that changes in placental nutrient transport, especially lipid transport and energy metabolism [43,44], directly contribute to altered foetal growth [25]. However, few of the proteins participating in lipid and energy transport and metabolism were significantly differentially expressed in MS gilts. Therefore, similar foetal weights were found at different uterine positions in MS gilts.

## Conclusion

Our study has described the relationship between foetal weight and uterine position and has revealed differences between the LW and MS breeds during pregnancy. This study provides the first evidence of differences in the proteomes between placentas located at the utero-tubal junction and those located at the cervix in both LW and MS gilts. Placental lipid and energy metabolism may be a crucial determinant of differences in foetal weight. The results of this study provide an opportunity to improve our understanding of placental efficiency. In a follow-up experiment, we will explore the further mechanistic studies to determine the role of those proteins found in proteomic approach.

## Supporting Information

S1 Fig The correlation of fold change between iTRAQ and MRM. A: the correlation of Large White gilt; B: the correlation of Meishan gilt.
(TIF)

S1 Table Primer sequences of the target and reference genes. (DOC)

S2 Table Differentially expressed proteins in the placenta in Large White and Meishan gilts. Proteins with a threshold of >1.2- or <0.83—fold difference in abundance with P values <0.05.
(XLS)

S3 Table Details of the MRM analysis. (DOC)

S4 Table List of all proteins (n = 2047) identified in the study. (XLS)

S5 Table MRM validation of differentially expressed proteins in LW and MS gilts. (DOC)
