# Supplementary figures and images for: Detection of Placental Proteomes at Different Uterine Positions in Large White and Meishan Gilts on Gestational Day 90
Source: PLoS One. 2016 Dec 9;11(12):e0167799. doi: 10.1371/journal.pone.0167799 (PMC5147991; doi:10.1371/journal.pone.0167799)

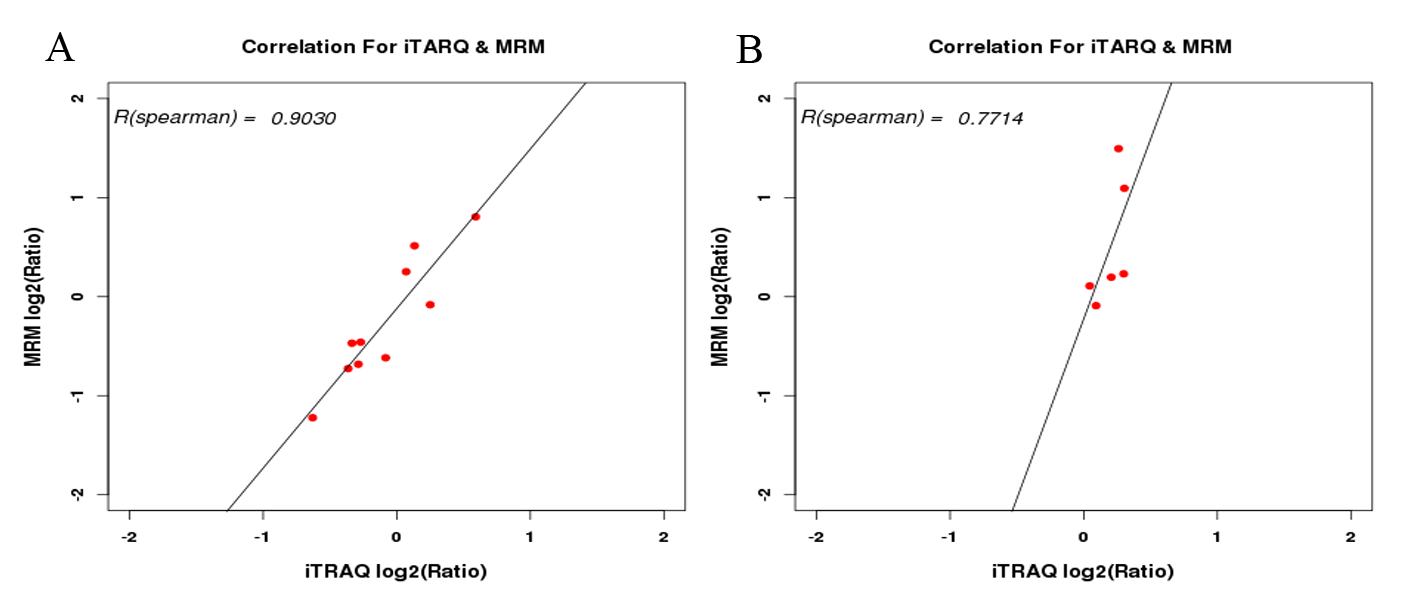

Supplement: S1 Fig — A: the correlation of Large White gilt; B: the correlation of Meishan gilt. (TIF) [file pone.0167799.s001.tif]
