# Supplementary material for: Detection of Placental Proteomes at Different Uterine Positions in Large White and Meishan Gilts on Gestational Day 90
Source: PLoS One. 2016 Dec 9;11(12):e0167799. doi: 10.1371/journal.pone.0167799 (PMC5147991; doi:10.1371/journal.pone.0167799)
Supplement: S1 Table — (DOC) [file pone.0167799.s002.doc]

| Genes | Primer | Sequence (5´→3´) | Products | Accession no |
| --- | --- | --- | --- | --- |
| GLUT1 | Forward | TCCTTCAGCCAGCAGTGATG | 125 | XM_005665507.1 |
|  | Reverse | AGCGTGGGATGTGGGTAAAG |  |  |
| GLUT3 | Forward | GGTGTAAAATCTGGAGGCTGGA | 155 | XM_003355585.3 |
|  | Reverse | GATGACTCCAGTGTTGTAGCCA |  |  |
| SLC7A4 | Forward | TTCTTCCTCTGGCTGGTGAT | 109 | NM_001243816.1 |
|  | Reverse | CCACATAGCGTGTGATGGTC |  |  |
| SLC27A1 | Forward | CTCATCTACGGGCTGATGGT | 108 | JN713898.1 |
|  | Reverse | GATCTCCCCGATGTACTGGA |  |  |
| ACADVL | Forward | TCCCCAAGACTCCATCAAAG | 121 | AB527055.1 |
|  | Reverse | ACCTCTTATCACCGCTGGAA |  |  |
| NDUFV2 | Forward | GAACTCAAGGCTGGCAAAAT | 117 | DQ629162.1 |
|  | Reverse | CACACCAAACCCAAGTCCT |  |  |
| ME1 | Forward | ATAGGAGTTGCTGCGATTGG | 115 | X93016.1 |
|  | Reverse | ATTCTGCTTTGCTGGTTGGA |  |  |
| RPS23 | Forward | TTCTGCCATCAGGAAGTGTG | 142 | AY461380.1 |
|  | Reverse | ATGACCTTTGCGACCAAATC |  |  |
| β-actin | Forward | GGCGCCCAGCACGAT | 66 | DQ845171.1 |
|  | Reverse | CGATCCACACGGAGTACTTG |  |  |

**S1 Table. Primer sequences of the target and reference genes.**

GLUT1: Glucose transporter 1; GLUT3: Glucose transporter 3; SLC7A4: Cationic amino acid transporter 4; SLC27A1: Solute carrier family 27; ACADVL: Long-chain acyl-CoA synthetase ACSL; NDUFV2: NADH dehydrogenase [ubiquinone] flavoprotein 2; ME1: NADP-dependent malic enzyme; RPS23: 40S ribosomal protein S23.
