# Supplementary material for: Detection of Placental Proteomes at Different Uterine Positions in Large White and Meishan Gilts on Gestational Day 90
Source: PLoS One. 2016 Dec 9;11(12):e0167799. doi: 10.1371/journal.pone.0167799 (PMC5147991; doi:10.1371/journal.pone.0167799)
Supplement: S3 Table — (DOC) [file pone.0167799.s004.doc]

**MRM validation of differentially expressed proteins from iTRAQ**

A spectral library of MS/MS data was generated on a TripleTOF5600 (AB SCIEX, Foster City, CA) and searched using Mascot v2.3 (Matrix Science, UK) against with a Pig database (48278 entries). The date file was imported into Skyline software where a library was built. The peptides was selected for MRM method development according to the following criteria: (1) the peptides with unique sequence in the database; (2) a maximum m/z of peptide < 1250 (limination of quandrupole scan ),with a peptide length range 5-40 amino acids length; (3) without methionine in peptides;(4) with carbamidomethyl on cysteine and without variable modification in peptides; and (5) no missed cleavage of trypsin. We initially monitored 6 transitions per peptide to ensure specificity with the criteria that >5 y-ions with the same elution profile and in the same ratios as the spectral library. The predicted retention time of targeted peptides was observed with an IRT strategy. A pooled peptides digested as described was performed preliminary SRM assays used to determine where these proteins were detected.

Samples were digested as described and spiked with 50 fmol of β-galactosidase for data normalization. MRM analyses were performed on a QTRAP5500 mass spectrometer (AB SCIEX, Foster City, CA) equipped with LC-20AD nanoHPLC system (Shimadzu, Kyoto, Japan). The Mobile phase consisted of solvent A, 0.1%aqueous formic acid and solvent B, 98% acetonitrilewith0.1% formic acid. Peptides were separated on a C18 column (0.075x 150mm column,3.6 μm) at 300 nL/min, and eluted with a gradient of 5%-30% solvent B for 38 min, 30%-80% solvent B for 4 min, and maintenance at 80% for 8 min. For the QTRAP5500 mass spectrometer, spray voltage of 2400 V, nebulizer gas of 23 p.s.i., and a dwell time of 10 ms were used. Multiple MRM transitions were monitored using unit resolution in both Q1 and Q3 quadrupoles to maximize specificity. Each MRM transition had a minimum dwell time of 10ms. We use Skyline software to integrate the raw file generated by QTRAP 5500 (SCIEX, Framingham, MA, USA). We use an IRT strategy to define a chromotography of a given peptide against a spectral library. All transitions for each peptide were used for quantitation unless interference from the matrix was observed. A spiked of β-galactosidase is used for lable free data normalizaiton. We use MSstats with the linear mixed-effects model the P values were adjusted to control the FDR at a cutoff of 0.05. All proteins with a P-value below 0.05 and a fold change larger than 1.5 are considered significant.
