# Supplementary material for: Detection of Placental Proteomes at Different Uterine Positions in Large White and Meishan Gilts on Gestational Day 90
Source: PLoS One. 2016 Dec 9;11(12):e0167799. doi: 10.1371/journal.pone.0167799 (PMC5147991; doi:10.1371/journal.pone.0167799)
Supplement: S5 Table — (DOC) [file pone.0167799.s006.doc]

**S5 Table MRM validation of differentially expressed proteins in LW and MS gilts**.

| Accession number | Protein name | LW | | |  | MS | | |
| --- | --- | --- | --- | --- | --- | --- | --- | --- |
|  |  | Fold change  (iTRAQ) | Fold change  (MRM) | p-value^*^ |  | Fold change (iTRAQ) | Fold change (MRM) | p-value^*^ |
| gi|198282077 | cytochrome P450 3A46 | 0.85 | 0.70 | 0.64 |  | 1.24 | 3.40 | 0.26 |
| gi|281427372 | ferrochelatase | 1.08 | 1.54 | 0.08 |  | 1.31 | 2.13 | 0.03 |
| gi|311247118 | NADH dehydrogenase [ubiquinone] flavoprotein 1 | 1.12 | 1.61 | 0.04 |  | 1.06 | 1.37 | 0.16 |
| gi|335308671 | 60S ribosomal protein L23a | 1.26 | 1.67 | 0.11 |  | 1.12 | 1.14 | 0.76 |
| gi|350590439 | acyl-CoA synthetase family member 2 | 1.75 | 1.49 | 0.05 |  | 0.98 | 1.96 | 0.04 |
| gi|7387634 | trifunctional enzyme subunit alpha | 1.59 | 2.33 | 0.00 |  | 1.15 | 1.49 | 0.04 |
| gi|753704324 | ATP synthase, H+ transporting | 1.23 | 1.39 | 0.11 |  | 1.06 | 0.94 | 0.82 |
| gi|927177157 | 40S ribosomal protein S25 | 1.18 | 1.37 | 0.11 |  | 1.26 | 1.17 | 0.56 |
| gi|927135975 | N-acetylgalactosamine-6-sulfatase | 1.04 | 0.94 | 0.79 |  | 1.00 | 1.38 | 0.28 |
| gi|937575546 | fucosidase | 1.21 | 1.19 | 0.56 |  | 1.33 | 2.82 | 0.02 |

LW: Large White gilts; MS: Meishan gilts.
